# Successful replantation of 2 digits in a patient with thrombocytosis after splenectomy: A case report: Erratum

**DOI:** 10.1097/MD.0000000000011515

**Published:** 2018-07-06

In the article, “Successful replantation of 2 digits in a patient with thrombocytosis after splenectomy: A case report”,^[1]^ which appeared in Volume 97, Issue 22 of *Medicine*, the author's names were spelled incorrectly in the author contributions and should appear as:

Conceptualization: Jae Ha Hwang.

Data curation: Dong Wan Kim.

Methodology: Dong Wan Kim.

Writing – original draft: Jae Ha Hwang.

Writing – review & editing: Kwang Seong Kim, Sam Yong Lee.
